# Case Report: Prolonged Survival Following EGFRvIII CAR T Cell Treatment for Recurrent Glioblastoma

**DOI:** 10.3389/fonc.2021.669071

**Published:** 2021-05-07

**Authors:** Joseph S. Durgin, Fraser Henderson, MacLean P. Nasrallah, Suyash Mohan, Sumei Wang, Simon F. Lacey, Jan Joseph Melenhorst, Arati S. Desai, John Y. K. Lee, Marcela V. Maus, Carl H. June, Steven Brem, Roddy S. O’Connor, Zev Binder, Donald M. O’Rourke

**Affiliations:** ^1^ Glioblastoma Translational Center of Excellence, The Abramson Cancer Center, Perelman School of Medicine at the University of Pennsylvania, Philadelphia, PA, United States; ^2^ Center for Cellular Immunotherapies, University of Pennsylvania, Philadelphia, PA, United States; ^3^ Department of Pathology & Laboratory Medicine, Perelman School of Medicine at the University of Pennsylvania, Philadelphia, PA, United States; ^4^ Department of Neurosurgery, Medical University of South Carolina, Charleston, SC, United States; ^5^ Department of Radiology, Division of Neuroradiology, Perelman School of Medicine at the University of Pennsylvania, Philadelphia, PA, United States; ^6^ Division of Hematology/Oncology, Perelman School of Medicine at the University of Pennsylvania, Philadelphia, PA, United States; ^7^ Department of Neurosurgery, Perelman School of Medicine at the University of Pennsylvania, Philadelphia, PA, United States; ^8^ Cellular Immunotherapy Program, Massachusetts General Hospital Cancer Center and Harvard Medical School, Boston, MA, United States

**Keywords:** CAR T cell therapy, glioblastoma, EGFRvIII, recurrent glioblastoma (rGBM), CAR (chimeric antigen receptor), perfusion imaging

## Abstract

Autologous chimeric antigen receptor (CAR) T cells targeted to epidermal growth factor receptor variant III (CAR T-EGFRvIII) have been developed and administered experimentally to treat patients with IDH1 wildtype recurrent glioblastoma (rGBM) (NCT02209376). We report the case of a 59-year-old patient who received a single peripheral infusion of CAR T-EGFRvIII cells and survived 36 months after disease recurrence, exceeding expected survival for recurrent glioblastoma. Post-infusion histopathologic analysis of tissue obtained during a second stage surgical resection revealed immunosuppressive adaptive changes in the tumor tissue as well as reduced EGFRvIII expression. Serial brain imaging demonstrated a significant reduction in relative cerebral blood volume (rCBV), a measure strongly associated with tumor proliferative activity, at early time points following CAR T treatment. Notably, CAR T-EGFRvIII cells persisted in her peripheral circulation during 29 months of follow-up, the longest period of CAR T persistence reported in GBM trials to date. These findings in a long-term survivor show that peripherally administered CAR T-EGFRvIII cells can persist for years in the circulation and suggest that this cell therapy approach could be optimized to achieve broader efficacy in recurrent GBM patients.

## Introduction

Autologous T cells redirected with chimeric antigen receptors (CARs) targeting EGFRvIII represent an investigational treatment paradigm for glioblastoma (GBM), the most common and aggressive adult primary brain malignancy (1). Neither surgery, chemotherapy nor radiation can completely control the disease, and second-line therapies for recurrence remain limited. Though median survival is 9 months after recurrence (2), we report a patient treated with CAR T-EGFRvIII who survived 36 months after disease recurrence.

CARs are synthetic surface receptors, combining artificial extracellular single chain antibody fragments for target cell recognition and intracellular T-cell activation and co-stimulation domains [(3), STM]. CAR T cells have proven highly efficacious against B cell malignancies, leading in 2017 to the FDA approval of two CD19 antigen-specific CAR T cell products in refractory B cell cancers. These results have raised expectations for CAR T in other cancers including GBM (4). At our institution, O’Rourke and colleagues conducted the first-in-human trial (NCT02209376) of autologous T cells redirected to EGFRvIII for rGBM (5). At the time of publication of that trial, three patients remained alive, including one who had remained alive and well without further therapy for more than 18 months. Here we present detailed analysis and an updated clinical course of that patient, with new data on histopathology, survival, duration of CAR T persistence, immunosuppressive responses in the tumor tissue, and perfusion MRI metrics (5). This analysis of a patient who received CAR T for rGBM suggests that a single peripheral infusion of CAR T cells may have on-target anti-tumor activity.

## Case Description

A previously healthy 58-year-old right-handed woman presented to an outside institution for difficulty reading and writing. MRI revealed a 23 cm (4) (4.0 cm max. diameter) contrast-enhancing lesion in the left posterior temporal lobe. She underwent near complete resection without complication, and histopathology confirmed the diagnosis of GBM with positive O (6)-methylguanine-DNA methyltransferase (MGMT) methylation, negative mutant IDH1 (R132H), and 60% EGFRvIII positivity. She completed three months of standard-of-care chemoradiation therapy (Stupp Protocol) and was then referred to the University of Pennsylvania with Karnofsky Performance Status (KPS) of 90, no focal neurologic deficits, and no steroid requirement. Surveillance MRI with dynamic susceptibility contrast (DSC) perfusion imaging revealed elevated rCBV suggesting tumor recurrence 6 months after initial resection (7). She was enrolled in our EGFRvIII-directed CAR T for recurrent GBM trial (NCT02209376).

**Figure 1:**
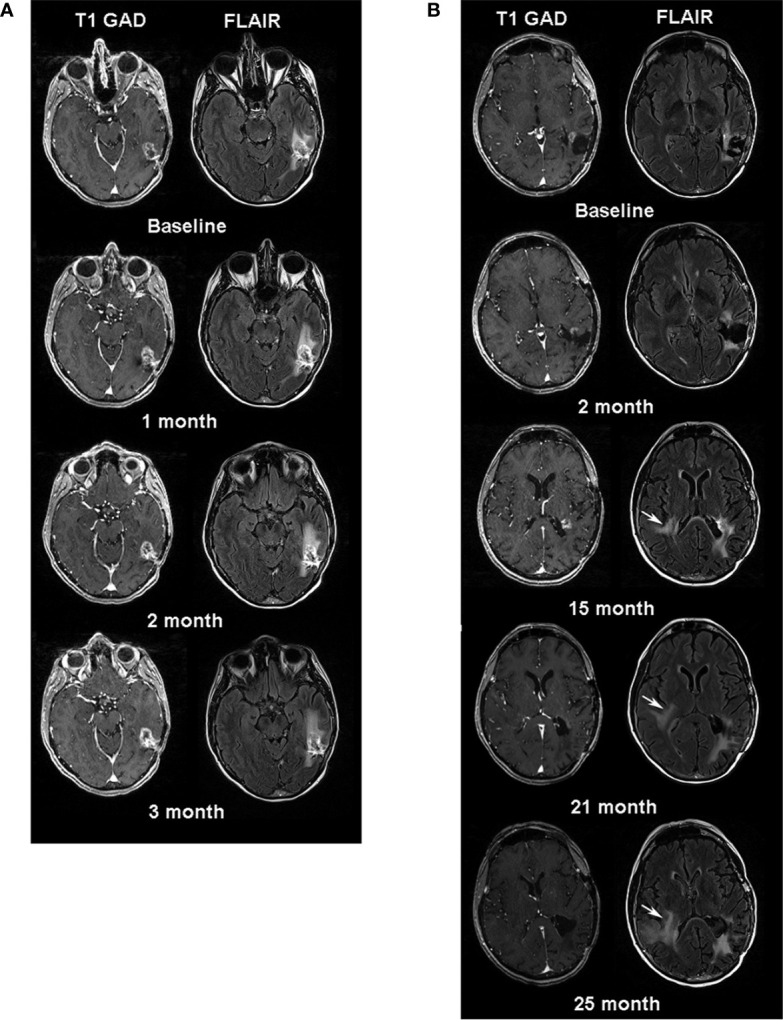
**(A)** Serial MR images one week before (baseline) and three months after CAR T-EGFRvIII infusion. Left sided images are axial post contrast T1 weighted images and right sided images are axial FLAIR images. Note, minimal increase in size of the enhancing lesion in the left temporal lobe at follow-up time-points. **(B)** Serial MRI examinations after the patient’s second surgery. The baseline is one month after her second surgery. White arrows at 15, 21- and 25-month follow-up periods indicate the increasing signal abnormality in the right periventricular region on FLAIR images.

Twenty-six days after leukapheresis, she underwent intravenous infusion of 9.2 x 10^7^ autologous EGFRvIII-directed CAR T cells. Two weeks prior to infusion, she had reported worsening pressure headaches, an increase in word-finding difficulty, and a “whooshing” sound in her left ear. These symptoms improved with a one-week steroid taper. On post-infusion day 7, she reported mild flu-like symptoms, including arthralgia, myalgia, and headache. She was managed conservatively with acetaminophen, which relieved her symptoms. Three months after CAR T cell infusion, the patient experienced increasingly severe headaches, requiring the re-initiation of dexamethasone. MRI demonstrated increased size of the enhancing lesion with increasing FLAIR abnormality, concerning for tumor progression vs. treatment response, the latter supported by decreased tumor relative cerebral blood volume (rCBV) (**Figures 1A, B**). To address her clinical complaints, and to potentially guide further therapy with tissue diagnosis, the patient underwent a second craniotomy on post-CAR T day 104. The pathology was consistent with recurrent GBM. She was discharged home with KPS 80 on post-operative day 2. At her post-operative visit, she reported difficulty with reading, consistent with a partial (quadrant) visual field deficit.

**Figure 2:**
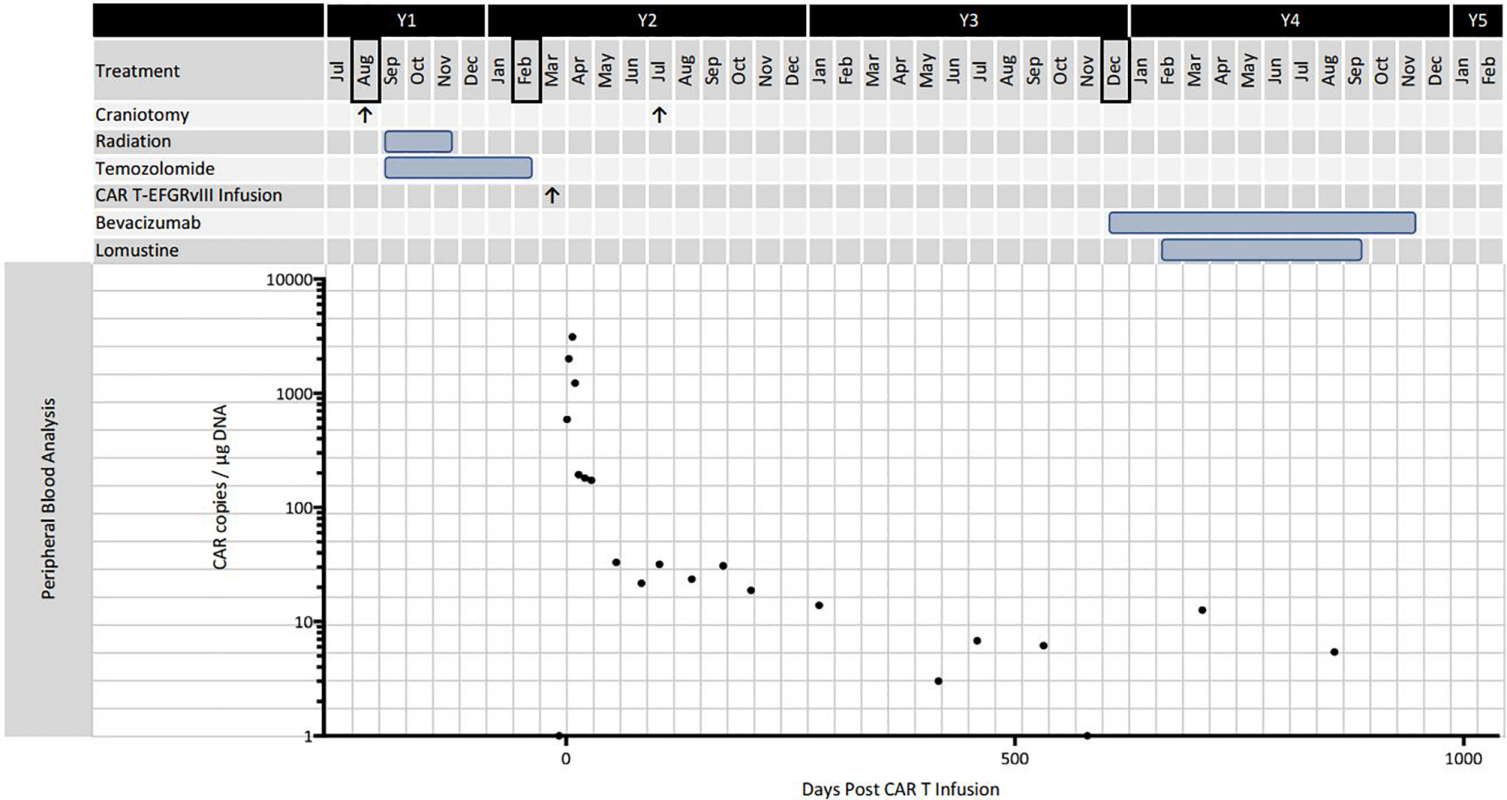
Timeline of disease progression and treatment. Black bordered months indicate diagnosis or definitive progression on surveillance MRI. The graph shows CAR T-EGFRvIII levels in the peripheral blood as measured by qPCR.

The patient enjoyed good functional status and received no additional chemotherapy for 18 months following her second operation (**Figure 2**). After 15 months, serial MRIs revealed gradual tumor progression (arrows, **Figure 1B**). At 32 months from CAR T infusion, she developed a methicillin sensitive *S. aureus* osteomyelitis in her right lower extremity and required operative debridement. After surgery she had a rapid, unexpected decline, never fully regaining her mental status, and in accordance with her and her family’s wishes she was transferred to hospice care. Her overall survival was 36 months from the date of initial tumor recurrence, and 34 months from CAR T infusion.

## Methods

### Immunohistochemistry

Five-micron sections of formalin-fixed paraffin-embedded tissue were stained using antibodies against PD1 (Clone NAT105; abcam 52587 1:40 dilution), Foxp3 (clone 206D, Biolegend 320102 1:100 dilution) and EGFR VIII (Cell Signaling 64952, 1:100. Staining was performed on a Leica Bond™ instrument using the Bond Polymer Refine Detection System (Leica Biosystems DS9800). Heat-induced epitope retrieval was done for 20 minutes in ER1 solution (Leica Biosystems AR9961) for PD1 and ER2 solution (Leica Biosystems AR9640) for Foxp3 and EGFR. To quantify IHC results, we used Fiji (ImageJ) to deconvolute hematoxylin from 3,3′-diaminobenzidine staining, followed by thresholding of signal to positive cells to create a binary image. The resulting output was passed through the watershed algorithm followed by automated particle analysis to determine the percent positivity by area.

### Radiographic Analysis

The DTI maps (MD, FA), CBV, Cho, Cr maps and FLAIR images were co-registered to contrast-enhanced T1-weighted images. A semi-automatic segmentation approach was used to generate a mask from the enhancing region of the neoplasm. The CBV values were normalized to the contra-lateral normal white matter to obtain relative CBV (rCBV). The median values of DTI parameters, rCBV and Cho/Cr from the enhancing regions of the neoplasms were estimated at each time point. Additionally, the top 90th percentile rCBV values were computed and reported as rCBVmax. The percent changes for each parameter (MD, FA, rCBV, rCBVmax and Cho/Cr) between the baseline and the subsequent scans (N) were calculated as (N – baseline)/baseline × 100.

### Study Approval

All experiments were performed in accordance with the approval by the Abramson Cancer Center Clinical Trials Scientific Review Committee, the Penn Institutional Biosafety Committee, and Institutional Review Board.

## Results and Discussion

**Figure 3:**
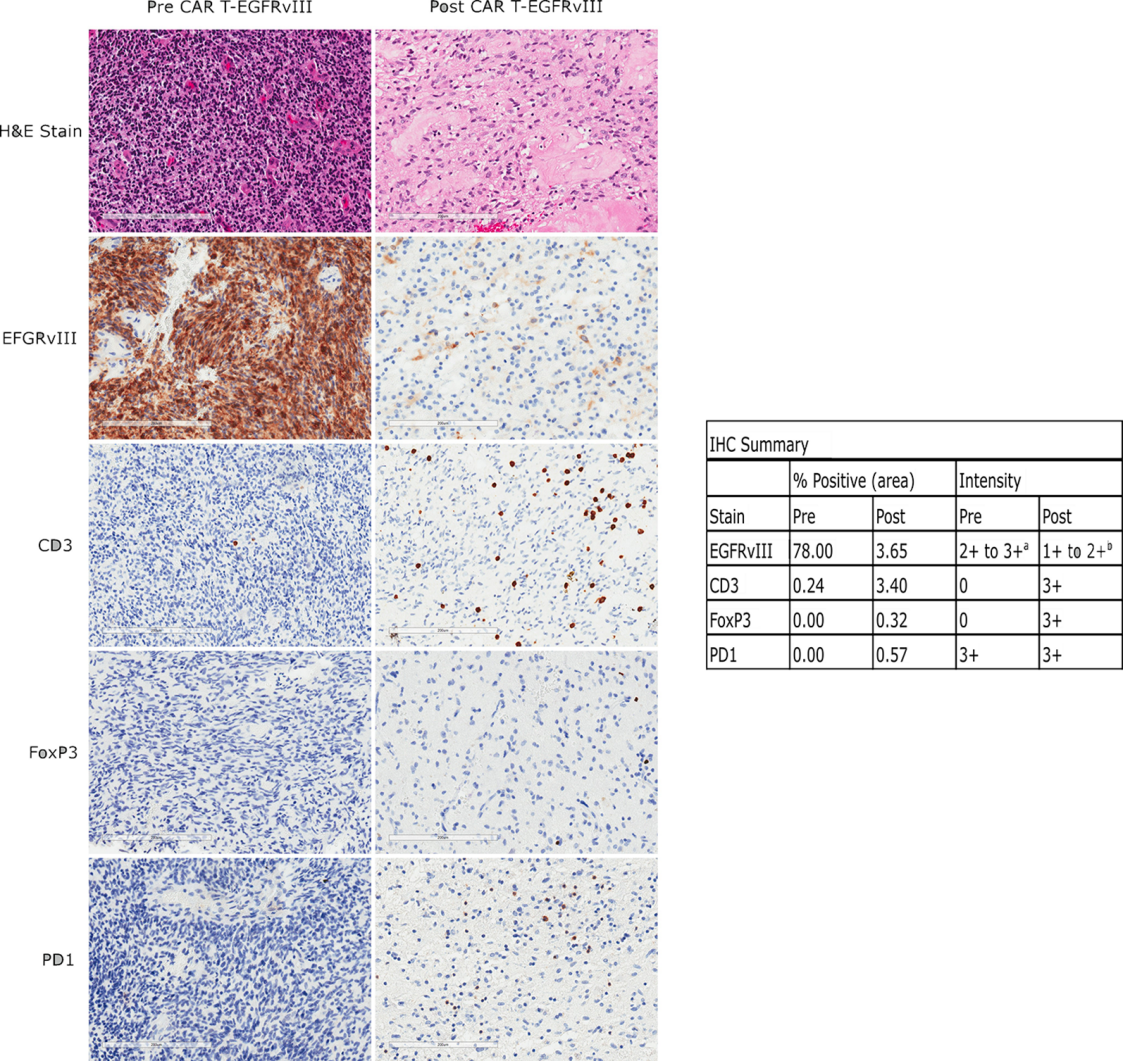
Immunohistochemical (IHC) analysis of EGFRvIII, CD3, FoxP3, and PD1 expression in the patient’s tumor before and after CAR T infusion. The left panels show tissue from her original surgery, prior to any treatment. The right panels show tissue from her surgery 104 days after CAR T infusion. Scale bars are 200 µm. Percent positivity by area was calculated by ImageJ as described in the methods, and staining intensity is by pathologist assessment. a: Pre-treatment fields also contain scattered 1+ EGFRvIII positive cells of unknown type. b: The positive cells are of undetermined lineage.

Histopathological examination of the tumor tissue obtained 104 days after CAR T-EGFRvIII infusion revealed recurrent and residual malignant glioma on a background of treatment related changes (**Figure 3**). Immunohistochemistry (IHC) showed a reduction in EGFRvIII expression from 78.0% to 3.7% by tumor area (**Figure 3**), suggesting possible on-target activity against antigen-expressing tumor cells. As published in the original clinical trial, we also employed an EGFRvIII-targeted RNA sequencing assay to determine trial eligibility (5). This assay determines the ratio of EGFRvIII transcripts (defined by deletion of exons 2 through 7) to total EGFR transcripts. For this patient, the RNA sequencing assay showed that EGFRvIII transcripts decreased to 13% compared to 60% in the pre-infusion specimen (5, 6). The post-CAR T tumor also had a more prominent T cell infiltrate as measured by CD3 staining (**Figure 3**). Deep sequencing of the *TCRβ CDR3* gene in the tumor tissue demonstrated an increase in the diversity of the T-cell clones, suggesting that a polyclonal T cell infiltrate had been recruited to the tumor (5). Peripheral blood samples detected CAR T-EGFRvIII cells throughout the 29 months of follow-up (**Figure 2**), indicating that the product engrafted successfully in the patient and recirculated through the peripheral blood (5).

**Figure 4:**
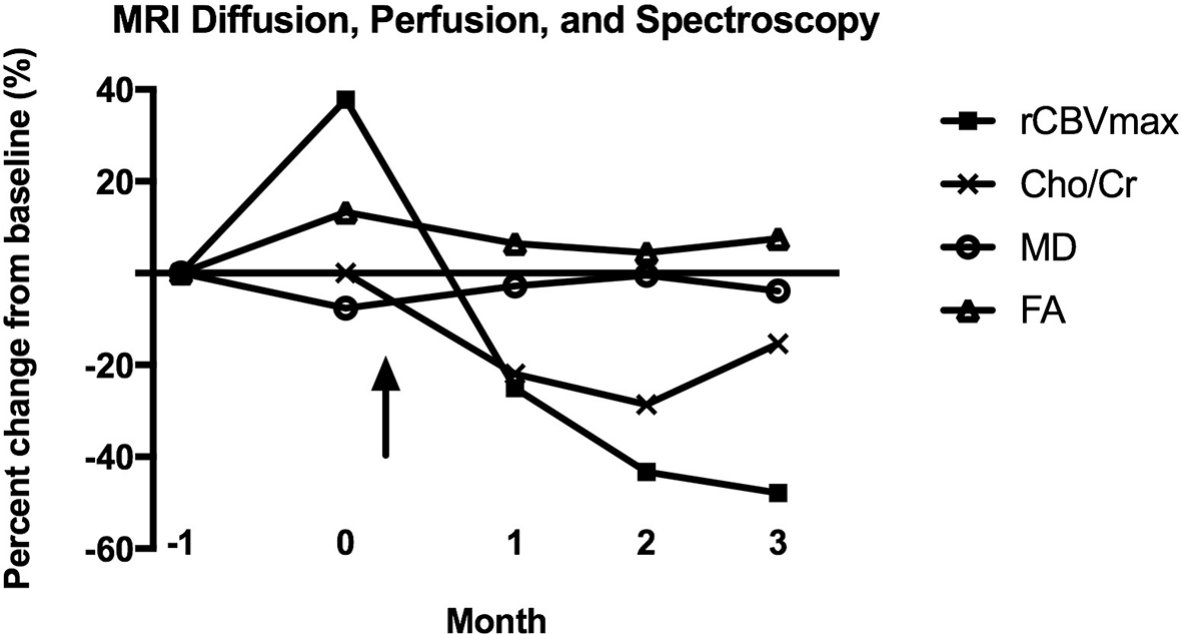
Percent change from baseline for MRI diffusion (MD, FA), perfusion (rCBVmax), and spectroscopy (Cho/Cr) parameters after CAR T-EGFRvIII infusion. No MR spectroscopy was performed at month -1, so the baseline for Cho/Cr is month 0. The arrow indicates the date of CAR T-EGFRvIII infusion. MD, mean diffusivity; FA, fractional anisotropy; rCBVmax, maximum relative cerebral blood volume; Cho/Cr, choline/creatine ratio.

At three years from initial tumor recurrence, this patient’s greater than expected survival may have been due to a combination of factors. Positive prognostic factors included a good performance status, a unifocal lesion, and no initial steroid dependence (8). Negative prognostic factors included the lack of IDH1 mutation, unmethylated MGMT on the recurrent tumor, and the large tumor volume (8). Based on the EORTC meta-analysis of clinical trials for rGBM, patients with similar prognostic features have a median overall survival on the order of 8 months from recurrence (8).

In a previously published case report, one patient receiving IL13Rα2-targeted CAR T experienced a complete, though transient, radiographic response for his multifocal rGBM (9). When the patient’s tumor did recur after 7.5 months, pathology showed decreased expression of the IL13Rα2 antigen. That case was also notable for evidence of recruitment of endogenous host immunity, with both CAR-expressing T cells and mixed endogenous immune cells present in the cerebral spinal fluid following treatments. In our case, the reduced expression of EGFRvIII antigen and increase in the number and diversity of T cell clones support trafficking of clones in the infusion product and suggest a local specific immune response related to antigen editing (5). Both the presented case and the IL13Rα2 study suggest that recruitment of a non-CAR T host immune infiltrate may constitute part of the therapeutic response to CAR T treatment.

The post-infusion tumor tissue in the current study demonstrated anti-inflammatory adaptations. In particular, the post-CAR T specimen showed a moderate level of programmed cell death protein 1 (PD-1) staining, whereas the baseline tumor had almost no staining (**Figure 3**) (5). Interestingly, this patient’s post-CAR T tissue did not demonstrate increases in regulatory T cell (Treg) markers (**Figure 3**), in contrast to other patients in the trial, possibly due to her second stage craniotomy occurring 104 days after CAR T treatment. The four patients with surgery within 13 days of infusion showed an increase in Treg markers, while no patients with later surgeries did so. The timing of and durability of expression of anti-inflammatory adaptive changes following CAR T treatment should be investigated further.

The lack of noninvasive biomarkers for anti-tumoral response remains a major problem in the surveillance of patients undergoing CAR T trials. Conventional MRI may be unreliable for assessing tumor progression post immunotherapy, as the inflammatory changes that accompany immunotherapies may lead to disruption of the blood brain barrier and resultant increasing areas of contrast enhancement and worsening FLAIR signal abnormality (10–12). While still investigational, advanced imaging modalities such as diffusion, perfusion and MR spectroscopy may be valuable in assessing CAR T treatment response. For this patient, the reduction in rCBV at early time points following CAR T treatment merits attention (**Figure 4**), as other studies have correlated rCBV to tumor biologic activity (13, 14). The rCBV metric assesses microvascular volume, an indirect measure of tumor angiogenesis (15). A prospective study of 53 glioma patients noted near perfect correspondence of tumor grading by rCBV versus histopathology, with a significant correlation between the mitotic index and rCBV values (13). In a trial of a dendritic cell vaccine in GBM, there were higher rCBV values in patients who progressed compared to those with stable disease (14).

CAR T cells detectable by qPCR persisted at 29 months post infusion, the last sample collected, representing the longest interval of peripheral engraftment that has been reported in CAR T for GBM. This is comparable to the duration of engraftment for anti CD19 CAR T cells as reported in the ELIANA trial (16). A previous study using HER2 directed CAR T cells showed persistence at 12 months (17). The recent clinical trial by Goff et al. of EGFRvIII directed CAR T cells in seventeen patients was notable for one survivor at 59 months but no data on CAR T persistence past 9 months (18). In our patient, the peak level of CAR T cells detected in the peripheral blood occurred at approximately seven days, which is comparable to CD19 CAR T pharmacokinetics (16). This coincided with the patient’s mild systemic symptoms, suggesting a cytokine-driven immune response. The CAR T expansion and related inflammatory syndrome are evidence that T cell activation occurred. The authors of the IL13Rα2 trial noted a similar correspondence of flu-like symptoms and cytokine levels with CAR T treatment, although this happened more quickly in their trial, at day one or two post CAR T infusion (9). The more rapid CAR T expansion in the IL13Rα2 study was perhaps due to intra-tumoral and intraventricular locoregional delivery, which may have expedited the interaction of CAR T and tumor cells.

While this case suggests evidence for a CAR T therapeutic response in rGBM, increased efficacy may occur with the next generations of CAR T cell therapy. First, strategies targeting multiple antigens may overcome the dual challenges of heterogenous antigen expression and selective deletion. Second, the increase in PD-1 and other immunoregulators following CAR T are suggestive of adaptive changes that may allow tumors to evade the current generation of therapies (5). Approaches that combine CAR T with immune checkpoint blockade agents like PD-1 inhibitors may be one solution for potentiating CART persistence required to result in clinical efficacy. The combination of CAR T-EGFRvIII and PD-1 blockade is currently the focus of a clinical trial at our institution for newly diagnosed GBM (NCT03726515). Tumor-associated macrophages and microglia, accounting for as many as half of the cells in the GBM TME (19–21), also exert a significant immunoinhibitory function, and therapies such as CD40 agonists may re-educate macrophages to destroy tumor stroma and support T cell activation (22). Finally, strategies that deliver T cells or adjuvant treatments directly to the tumor or ventricular system may enhance efficacy (9). Through gene engineering, CAR T cells can be equipped with new features that address the challenges observed in the first generation of clinical trials. The responses seen to date suggest that CAR T therapy can be optimized to make a significant therapeutic impact in GBM.

## Data Availability Statement

The original contributions presented in the study are included in the article/supplementary material. Further inquiries can be directed to the corresponding author.

## Ethics Statement

The studies involving human participants were reviewed and approved by Hospital of the University of Pennsylvania IRB. The patients/participants provided their written informed consent to participate in this study. Written informed consent was obtained from the individual(s) for the publication of any potentially identifiable images or data included in this article.

## Author Contributions

JD, FH, MN, SM, and SH contributed to data acquisition and analysis and the writing of the manuscript. SL, JJM, AD, and JL contributed to data acquisition and the writing of the manuscript. DO’R, ZB, RO’C, MM, and CJ contributed to study design and the writing of the manuscript. All authors contributed to the article and approved the submitted version.

## Funding

This study was funded, in part, by the Glioblastoma Translational Center of Excellence within The Abramson Cancer Center at the University of Pennsylvania. The clinical trial NCT02209376 was funded by a partnership between Novartis and the University of Pennsylvania for the development of CAR T cells for cancer.

## Conflict of Interest

DO’R and ZB are inventors on patents related to CAR T cells that have been filed by the University of Pennsylvania. JJM consults with or serves on the board of directors of several companies developing CAR T technology. JM and SL are inventors of intellectual property related to CAR T cells that is licensed by the University of Pennsylvania to Novartis.

CHJ reports receiving grants from Tmunity Therapeutics and holds founders stock in Tmunity Therapeutics and DeCART Therapeutics. CHJ also receives personal income from BluesphereBio, Cabaletta, Carisma, Cellares, Celldex Therapeutics, Viracta Therapeutics, Ziopharm and WIRB-Copernicus Group as well as royalties from Novartis.

MCM is an inventor on patent applications related to CAR technology and has received licensing royalties from Novartis corporation.

The remaining authors declare that the research was conducted in the absence of any commercial or financial relationships that could be construed as a potential conflict of interest.
